# Supplementary figures and images for: Ginseng Saponin Enriched in Rh1 and Rg2 Ameliorates Nonalcoholic Fatty Liver Disease by Inhibiting Inflammasome Activation
Source: Nutrients. 2021 Mar 5;13(3):856. doi: 10.3390/nu13030856 (PMC7999915; doi:10.3390/nu13030856)

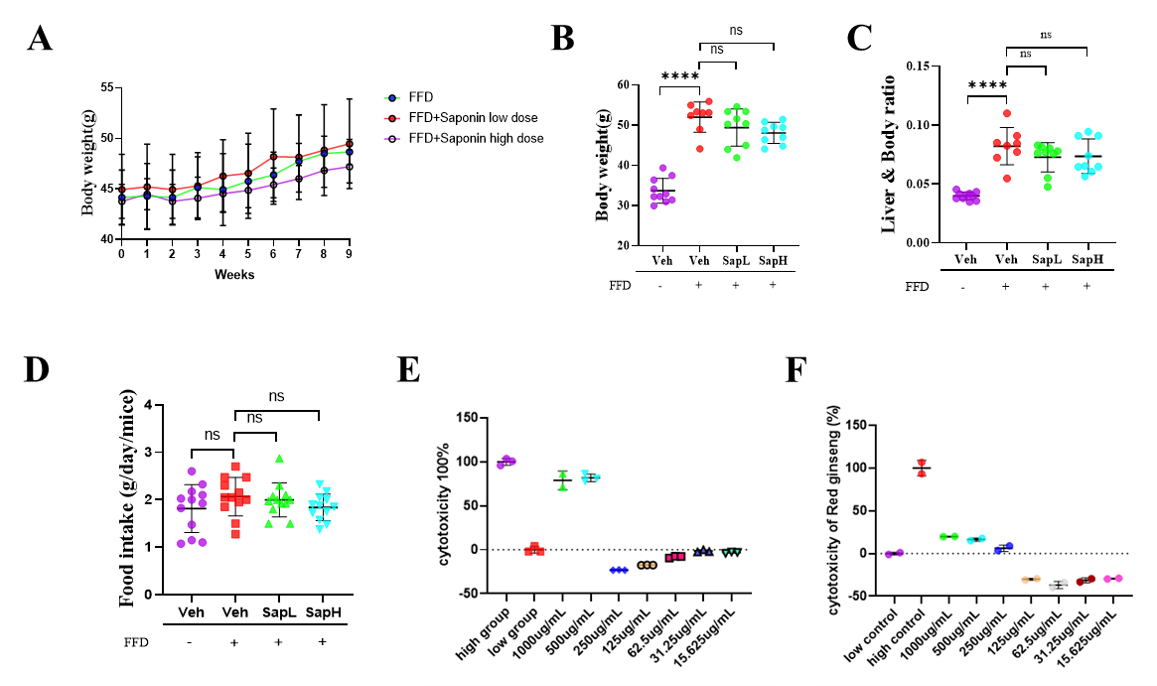

Supplement: Supplementary file 1 [file nutrients-13-00856-s001.zip › nutrients-1110074-Supplement Figure 1.tif]
